# Fullerene Aggregation in Thin Films of Polymer Blends for Solar Cell Applications

**DOI:** 10.3390/ma11112068

**Published:** 2018-10-23

**Authors:** Camilla Lindqvist, Ellen Moons, Jan van Stam

**Affiliations:** 1 Department of Engineering and Physics, Karlstad University, SE-651 88 Karlstad, Sweden; camilla.lindq@gmail.com (C.L.); Ellen.Moons@kau.se (E.M.); 2 Department of Engineering and Chemical Sciences, Karlstad University, SE-651 88 Karlstad, Sweden

**Keywords:** fullerene aggregation, organic solar cells, fluorescence, organic photovoltaics

## Abstract

We report on the effects of the film morphology on the fluorescence spectra for a thin film including a quinoxaline-based co-polymer (TQ1) and a fullerene derivative ([6,6]-phenyl-C_71_-butyric acid methyl ester—PC_70_BM). The ratio between the polymer and the fullerene derivative, as well as the processing solvent, were varied. Besides the main emission peak at 700 nm in the fluorescence spectra of thin films of this phase-separated blend, a broad emission band is observed with a maximum at 520–550 nm. The intensity of this emission band decreases with an increasing degree of mixing in the film and becomes most prominent in thicker films, films with high PC_70_BM content, and films that were spin-coated from solvents with lower PC_70_BM solubility. We assign this emission band to aggregated PC_70_BM.

## 1. Introduction

The interest in polymer based solar cells has grown rapidly during the last two decades. This technology shows several advantages, all related to its easy manufacturing by solution-based processes, compared to other solar energy harvesting technologies. Today single junction polymer solar cells of binary blends have reached a record efficiency of about 14% [1,2] and tandem cells even higher, above 17% [3].

The photoactive layer consists of a blend of an electron-donating polymer and an acceptor molecule. One of the most studied classes of acceptor molecules is fullerene derivatives, such as [6,6]-phenyl-C_61_-butyric acid methyl ester (PC_60_BM) or [6,6]-phenyl-C_71_-butyric acid methyl ester (PC_70_BM), but more recently non-fullerene acceptor molecules or polymers have increased in popularity. The donor and acceptor material are typically processed together from a chlorinated and/or aromatic solvent into a thin liquid film. The morphology of the donor/acceptor active layer is formed during deposition when the solvent evaporates and can later be altered through post-production treatments [4,5]. The morphology will affect the charge transfer from the donor to the acceptor, as well as the charge transport to the electrodes, and is consequently a crucial factor determining the solar cell performance [6,7,8,9]. 

**Figure 1:**
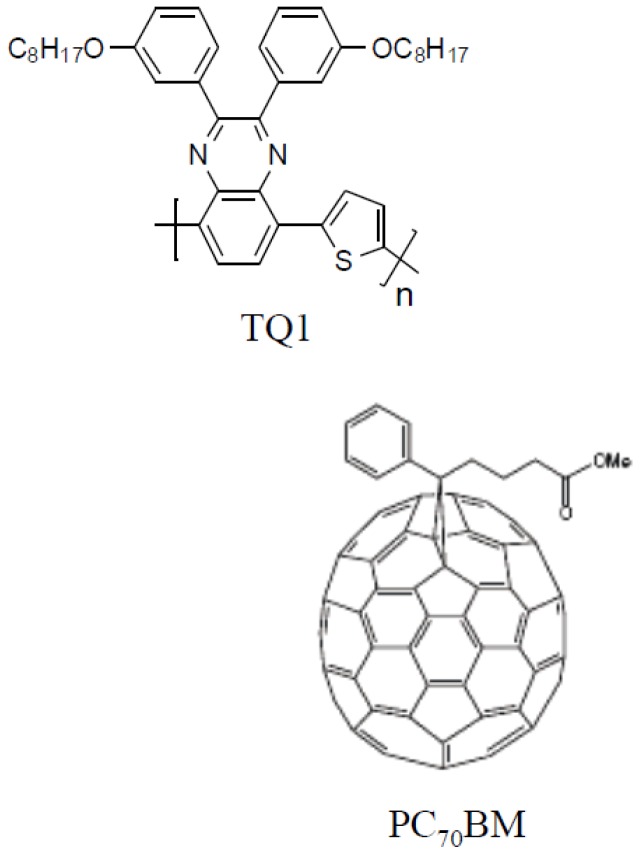
Chemical structure of a quinoxaline-based donor polymer (TQ1) and a [6,6]-phenyl-C_71_-butyric acid methyl ester (PC_70_BM).

When a polymer/fullerene blend phase separates, polymer-rich and fullerene-rich domains are formed. The degree of phase separation will depend on several factors, e.g., the solubility of the two materials in the solvent [10,11], their mutual miscibility [12,13], and the rate of drying [14,15]. The interaction between the fullerene derivative and the conjugated polymer, as for instance expressed by the Flory–Huggins interaction parameters, plays a significant role in the phase separation [12]. By varying the solvent used for deposition, the solubility and the drying kinetics are affected simultaneously, and the change in morphology is complicated to predict [10,16,17,18,19]. Microscopy techniques, such as atomic force microscopy (AFM), are very suitable to study domain structures of 50 nm up to several micrometres in diameter. For systems where liquid–liquid phase separation prevails, coarser domain structures will be achieved if the drying time is prolonged by e.g., slower spin-coating speeds [14,15] or solvent annealing. Blends of a quinoxaline-based donor polymer (TQ1) [20] and PC_70_BM (Figure 1) [10,21,22], have been shown to be a material combination that yields solar cell efficiencies up to 7% [23].

In order to access information about the structures on the molecular scale, it is of importance to combine microscopy techniques with others, e.g., fluorescence spectroscopy. Here, we report on the aggregation of PC_70_BM, as studied by fluorescence spectroscopy. Fluorescence spectroscopy has successfully been used for the characterisation of these [21,22] or other [24,25] blends previously. The optical properties are correlated with the phase separated domain structure as imaged by AFM.

## 2. Materials and Methods

PC_70_BM (purity 99%) was purchased from Solenne, Groningen, The Netherlands. TQ1 was prepared according to previously published procedures [20]. TQ1 had a number-average molecular weight of *M*_n_ ≈ 34,000 (polydispersity index ≈ 2.7) as measured with size exclusion chromatography with a polystyrene standard. Chlorobenzene (CB) and chloroform (CF) (analytical grade) were purchased from Merck (Kenilworth, NJ, USA), *ortho-*dichlorobenzene (*o*DCB) (analytical grade) was purchased from Sigma-Aldrich (St. Louis, MO, USA). All solvents were used as received. 

### 2.1. Solubility Measurements

Saturated solutions of PC_70_BM were prepared in the three solvents. After equilibration, the solutions were thoroughly centrifuged and an aliquot of known volume of the supernatant was withdrawn. The saturation concentrations were estimated by gravimetric analysis of the dry content after complete solvent evaporation. 

### 2.2. Thin Film Preparation

Thin films were spin-coated from CF (12.5 mg/mL), CB (20 mg/mL), or *o*DCB (25 mg/mL) solutions. Concentrations in brackets are the total material concentration in solution in which the ratio between TQ1 and PC_70_BM was altered. Solutions were gently heated prior to spin-coating, approximately 50 °C for CF and 60 °C for CB and *o*DCB. The spin-speed and concentrations were chosen to yield thin blend films of similar thickness [10]. The film thickness was approximately 90 nm (±10 nm), as measured by AFM, if nothing else is stated. The films were allowed to dry in ambient air. For fluorescence spectroscopy and AFM, silicon wafers were used as substrates, cleaned using the standard RCA method [26,27,28] without the final HF-etching step, so that a clean and hydrophilic surface is achieved. 

### 2.3. Steady-State Fluorescence Measurements

Steady-state fluorescence spectra were collected on a SPEX FL3-11 TAU fluorimeter, purchased from Gammadata (Uppsala, Sweden). Measurements were performed on thin films in front-face mode at a 22° angle relative to the incident excitation light in order to prevent reflected excitation light. The excitation wavelength was 380 nm. All samples were kept in the dark prior to the measurements and the spectra were recorded at room temperature and in ambient air.

### 2.4. Atomic Force Microscopy (AFM)

Images were collected in tapping mode with a Nanoscope IIIA Multimode AFM (Bruker, Billerica, MA, USA) using a TESPA-V2 n-doped silicon cantilever.

## 3. Results and Discussion

### 3.1. Solubility of PC_70_BM

The solubility of PC_70_BM in *o*DCB was determined by gravimetric analysis to be 66 mg/mL, compared to 56 mg/mL in CB and 34 mg/mL CF [22]. The vapour pressures of the solvents are 0.14 kPa, 1.5 kPa, and 26.3 kPa for *o*DCB, CB, and CF, respectively [29,30].

### 3.2. Fluorescence Measurements

**Figure 2:**
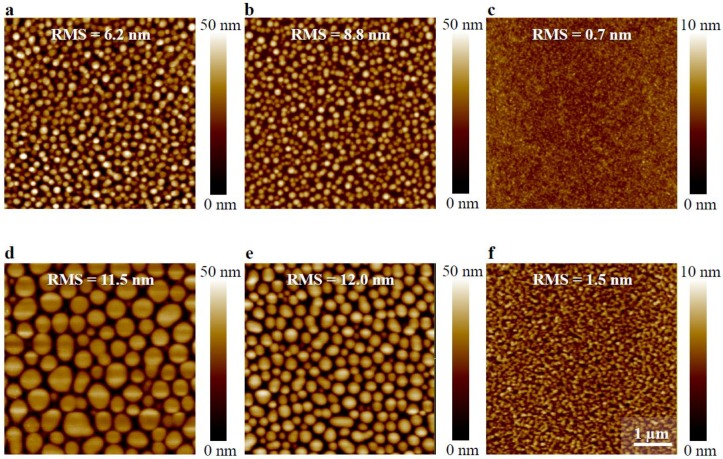
Atomic force microscopy (AFM) micrographs (5 × 5 µm) of thin films of 1:1 TQ1:PC_70_BM (**a**–**c**) and 1:3 TQ1:PC_70_BM (**d**–**f**) spin-coated from chloroform (CF) (**a**,**d**), chlorobenzene (CB) (**b**,**e**), and *ortho-*dichlorobenzene (*o*DCB) (**c**,**f**). Scale bar indicates 1 µm. Surface roughness (RMS), as well as the corresponding height scale, is added to each image.

**Figure 3:**
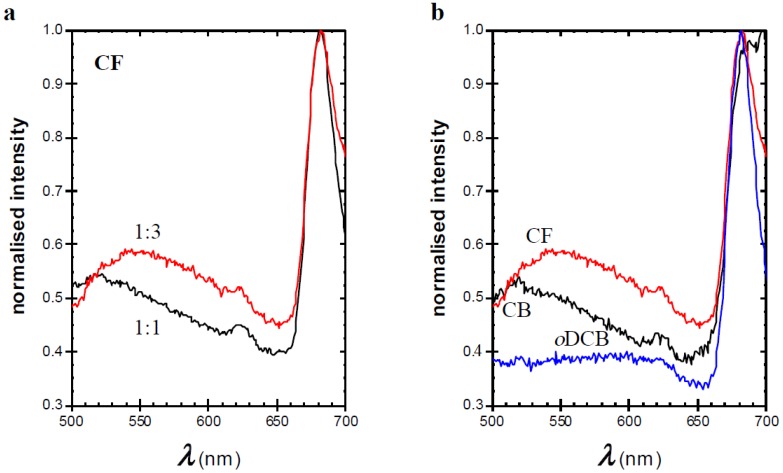
Fluorescence emission spectra of thin films measured with excitation wavelength (λ_ex_) = 380 nm of (**a**) 1:1 (black) and 1:3 (red) TQ1:PC_70_BM spin-coated from CF (**b**) 1:3 TQ1:PC_70_BM spin-coated from CF (red), CB (black), and *o*DCB (blue).

To study the relationship between morphology and optical properties, both the solvent for deposition and the ratio between TQ1 and PC_70_BM were altered. It has been shown earlier that the phase separated domains become larger when the fraction of PC_70_BM is increased in the blend [31] and that the domain size depends on the choice of solvent for films with equal thickness [10]. Hansson et al. have shown that the composition of the phase separated domains is rich in PC_70_BM and, by AFM imaging, that the domains are larger when CF is used as solvent than when *o*DCB is the solvent [10]. The AFM images shown in Figure 2 show that the phase-separated, PC_70_BM-rich domains become larger when a worse solvent for the fullerene derivative is used, in line with the earlier findings. This is more pronounced for the 1:3 blend than for the 1:1 blend in the series of decreasing PC_70_BM solubility, i.e., *o*DCB, CB, and CF. Emission spectra, shown in Figure 3, obtained upon excitation at 380 nm of the blend films show that apart from the main TQ1 emission peak at 700 nm [21,22], a very broad emission band with a maximum between 520 nm and 550 nm. For pure TQ1 in solution, this emission band is absent when TQ1 is dissolved in CF, CB or *o*DCB [22], which is also found for pure TQ1 films (spectrum not shown). The only emission in the visible region from TQ1 dissolved in those solvents is found around 650–700 nm, slightly blue-shifted in comparison with the emission from TQ1 films [21,22]. For TQ1:PC_70_BM blend films, we have shown that when the PC_70_BM-rich domains grow, the intensity of the emission band between 500 nm and 550 nm increases [22].

A similar trend in the emission peak as in the previous studies is observed if the amount of PC_70_BM was increased (Figure 3a) or if a worse solvent for PC_70_BM (CF) is used (Figure 3b). Further, the maximum of the 500–550 nm emission band shifts to longer wavelengths, i.e., from approximately 520 nm to 550 nm, shown in Figure 3. Cook et al. showed that films of pure PC_60_BM give rise to a broad emission band at approximately 500 nm, which is absent when the fullerene derivative is well distributed in a polystyrene matrix (1:7 fullerene/polystyrene) [31]. They assign this emission to the occurrence of aggregated PC_60_BM. In addition, Jamieson et al. observed this peak in films of blends with a high fullerene content in a semi-conducting polymer matrix relevant for solar cell applications [32]. The emission peak observed in the present PC_70_BM-based blends seems to be similar to the one reported for PC_60_BM. This series of results indicates that the 520–550 nm emission band in strongly phase-separated TQ1:PC_70_BM is likely to emanate from aggregated PC_70_BM, and that samples with larger domains include a larger portion of aggregated PC_70_BM. The nature of these aggregates is not clear from these results. Earlier literature suggests that nanocrystals of fullerenes can be formed upon spin-coating [33]. The shift from 520 nm to 550 nm in emission maximum is most likely due to a gradual development of the aggregates from a less organised cluster of PC_70_BM molecules (in films coated from CB and in films with a 1:1 ratio) to more compact aggregates in films with a 1:3 ratio or in films prepared from CF. For the present results, we cannot draw conclusions about the location of the PC_70_BM aggregates, even though it is safe to assume that they are formed inside the PC_70_BM-rich domains.

**Figure 4:**
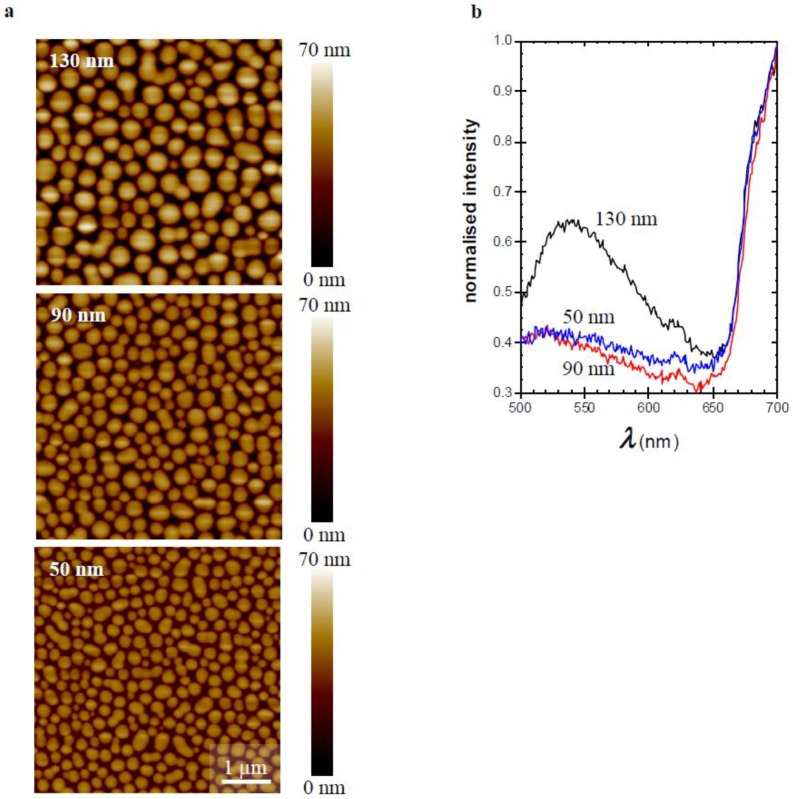
(**a**) AFM micrographs (5 × 5 µm) of thin films of 1:3 TQ1:PC_70_BM spin-coated from CB with the indicated film thicknesses. A height scale is added to each image. (**b**) Corresponding fluorescence emission spectra for films with indicated thicknesses.

To further underpin this interpretation, films of the 1:3 TQ1:PC_70_BM blend with different thicknesses (50–130 nm) were prepared [15] by changing the spin-coating speed (Figure 4). Thicker films, resulting from slower spin-coating, demonstrated larger PC_70_BM-rich domains as shown in the AFM images (Figure 4a). The corresponding emission spectra (Figure 4b) show that when the film thickness is increased from 90 nm to 130 nm, the intensity of the emission band increases and the emission maximum shifts from 520 nm to about 540 nm. Decreasing the film thickness from 90 nm to 50 nm does not have a significant effect on the emission band. This confirms that the domains have to reach a certain size before the aggregate emission becomes detectable. These results follow the trends presented by Bäcke et al., where they analysed thermally-induced aggregation of PC_60_BM by transmission electron tomography in blend films of TQ1:PC_60_BM. They showed that the PC_60_BM crystals grow in size and that the PC_60_BM rich domains start to develop in the upper part of the blend film, yielding crystals in this upper part [34].

## 4. Conclusions

In this study we have investigated the relationship between the detailed emission spectra analysis of TQ1:PC_70_BM blends and the morphology of these blends. Solvents with lower PC_70_BM dissolution capacity as well as an increased PC_70_BM concentration yield larger phase-separated domains. In these larger domains, PC_70_BM aggregates are likely to be formed, resulting in a broad and rather weak emission between 520 nm and 550 nm upon excitation at 380 nm. For better PC_70_BM solvents and at lower PC_70_BM concentrations, the fullerene derivative stays well-mixed with the polymer and no emission between 500 nm and 550 nm is detected. Further investigation by, e.g., high-resolution microscopy techniques is needed to determine the sizes and locations of these aggregates.

As aggregation of the components in the active layer of an organic solar cell is one of the major degradation paths, leading to a large drop in device efficiency, there is a need to detect and quantify this phenomenon. The methods used to measure aggregation in thin blend films are often both time-consuming and expensive. We have shown that fluorescence spectroscopy offers an easy and fast tool for this purpose, in comparison with other methods. More experimental work has to be done in order to further develop this method.
